# The complete mitochondrial genome of *Pomacea diffusa* (Gastropoda: Ampullariidae)

**DOI:** 10.1080/23802359.2017.1361348

**Published:** 2017-08-02

**Authors:** Suwen Liu, Qianqian Yang, Chao He, Xiaoping Yu

**Affiliations:** Zhejiang Provincial Key Laboratory of Biometrology and Inspection & Quarantine, College of Life Sciences, China Jiliang University, Hangzhou, China

**Keywords:** *Pomacea diffusa*, mitochondrial genome, phylogenetic analysis

## Abstract

In this study, we analyzed the complete mitochondrial genome of the spike-topped apple snail, *Pomacea diffusa* Blume, 1957 (Gastropoda: Ampullariidae). The mitochondrial genome of *P. diffusa* was 16,373 bp, consisting 13 protein-coding genes, 2 rRNAs, and 22 tRNAs and a non-coding region with a 12 bp repeat unit. There were 29 mitochondrial genes of *P. diffusa* are distributed on the H-strand, and the other eight tRNA genes encoded on the L-strand. The average AT content of 13 protein-coding genes was 68.7%. A phylogenetic analysis showed that there was a close relationship between *P. diffusa* and invasive apple snail species, *Pomacea canaliculata* and *Pomacea aff. maculata*.

## Introduction

*Pomacea diffusa* is a common pet apple snail species popular in aquarium trade, which has been introduced to Australia, Asia, and North America from South America (Hayes et al. 2008). However, unlike its congeners, such as *P. canaliculata* and *P. maculata*, *P. diffusa* is the only Ampullariidae species permitted to be transported between states by the United States Department of Agriculture (Gaston 2006; Rawlings et al. 2007). Owing to their similar shell morphology with a distinctive stair-like appearance on their whorls, *P. diffusa* was frequently referred to as *P. bridgesii* by mistakes (Cowie et al. 2006). In this study, we characterized the complete mitochondrial genome sequence of *P. diffusa* to contribute to further molecular and phylogenetic studies of this snail species.

Alive adults of *P. diffusa* were bought from an aquarium store, preserved in 95% ethanol and stored in −80 °C. The specimen was stored in China Jiliang University, China, under the code: 150302-IPS-PD-W-01. Genomic DNA was extracted from foot muscle using a Tiangen DNA extract kit (Tiangen Inc., Beijing, China) and obtained DNA eluted in 200 µl of sterile deionized water with a concentration of 69 ng/μl. We used the Illumina Hiseq 2500 platform to build up a genomic library and obtained the mitochondrial genome sequence of *P. diffusa*. The resultant reads were assembled and annotated using Geneious 7.0.6.

The complete mitochondrial genome of *P. diffusa* was of 16,373 bp long (GenBank accession number MF373586), thus 566 bp longer than *P. canaliculata* (Zhou et al. 2014) and 857 bp longer than *P. aff. maculata* (Yang et al. 2016). The complete mitochondrial genome has 37 genes, including 13 protein-coding genes, 22 putative tRNA genes, and two ribosomal RNA genes, and an AT-rich non-coding region. The nucleotide composition for the mitogenome was 29.9% of A, 39.6% of T, 14.3% of C, and 16.2% of G. Eight tRNA genes (*trnM, trnY, trnC, trnW, trnQ, trnG, trnE, trnT*) were encoded in the L-strand, and all other genes are encoded in the H-strand. All the protein-coding genes began with ATG as start codon. ATP6, ATP8, ND1-4, ND4L, ND6, COX3 genes were terminated with TAA as stop codon, while COX1, COX2, CYTB ended with TAG. The control region of *P. diffusa* was 806 bp, and the AT content was 73.8%. A 12bp-repeat unit (ATCTATACATAC) repeated 37 times, with 19 repeats encoded in the L-strand and 18 repeats encoded in the H-strand.

**Figure 1.**
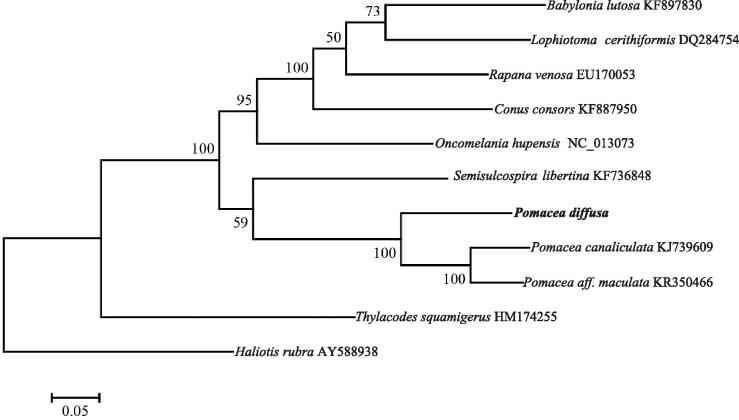
Maximum likelihood tree based on the nucleotide sequences of 13 protein-coding genes illustrating the phylogenetic position of *P. diffusa* among a subset of Caenogastropoda species. *Haliotis rubra* (Gastropoda: Vetigastropoda) was used as an outgroup.

To further validate the phylogenetic position of *P. diffusa*, we constructed maximum likelihood phylogenetic trees using MEGA 6.0 (Tamura et al. 2013) based on the nucleotide sequences of 13 protein-coding genes (Figure 1). We included mitochondrial genome sequences of other nine snail species in Caenogastropoda published in GenBank in the phylogenetic analyses. The phylogenetic position of *P. diffusa* was closely clustered with *P. canaliculata* and *P. aff. maculata*, which supported that the mitochondrial DNAs of *P. diffusa* were closely related to other species of *Pomacea*. The mitogenome of *P. diffusa* will provide essential data for further phylogenetic and evolutionary analysis for apple snails.
